# Application of Time-Series Analysis and Expert Judgment in Modeling and Forecasting Blood Donation Trends in Zimbabwe

**DOI:** 10.1177/23814683231222483

**Published:** 2024-01-18

**Authors:** Coster Chideme, Delson Chikobvu

**Affiliations:** Department of Mathematical Statistics and Actuarial Sciences, University of the Free State, Bloemfontein, South Africa; Department of Mathematical Statistics and Actuarial Sciences, University of the Free State, Bloemfontein, South Africa

**Keywords:** blood donation, time series, seasonality, forecasting, ETS, SARIMA

## Abstract

**Background.** Blood cannot be artificially manufactured, and there is currently no substitute for human blood. The supply of blood in transfusion facilities requires constant and timely collection of blood from donors. Modeling and forecasting trends in blood collections are critical for determining both the current and future capacity requirements and appropriate models of adequate blood provision. **Objectives.** The objective of this study is to determine blood collection or donation patterns and develop time-series models that can be updated and refined in predicting future blood donations in Zimbabwe when given the historical data. **Materials and Methods.** Monthly blood donation data for the period 2009 to 2019 were collected retrospectively from the National Blood Service Zimbabwe database. Time-series models (i.e., the Seasonal Autoregressive Integrated Moving Average [SARIMA] and Error, Trend and Seasonal [ETS]) models were applied and compared. The models were chosen because of their ability to handle the seasonality and other time-series components evident in the blood donation data. Expert opinions and experience were used in selecting the models and in making inferences in the analysis. **Results.** Time-series plots of blood donations showed seasonal patterns, with significant drops in blood donations in months associated with Zimbabwe’s school holidays (April, August, and December) and public holidays. During these holidays, there is a reduced number of school donors, while at about the same time, there is increasing blood demand as a result of road accidents. Model identification procedures established the $\mathrm{SARIMA}\;\left(1,1,2\right){\left(0,1,1\right)}_{12}$ model as the appropriate model for forecasting total blood donation in Zimbabwe. The results and forecasts show an upward trend in blood donations. According to the accuracy measures used, the SARIMA model outperforms the ETS model. **Conclusions.** Expert knowledge in the blood donation process, coupled with statistical models, can help explain trends exhibited in blood donation data in Zimbabwe. These findings help the blood authorities plan for blood donor campaign drives. The findings are key indicators of where to allocate more resources toward blood donation and when to collect more blood units. The increasing blood donation projections ensure a stable blood bank inventory in the near future.

**Highlights:**

- A SARIMA model can be used to predict the flow of blood donations in Zimbabwe.
- The seasonal blood donation pattern peaks in the months of March, June/July, and September.
- The donations troughs are in the months of April, August, December, and January. These are the months coinciding with school holidays in Zimbabwe.
- Both the SARIMA and ETS models provided similar forecasts, but measures of fit and expert knowledge gave a slight preference to the $\mathrm{SARIMA}\;\left(1,1,2\right){\left(0,1,1\right)}_{12}$ model in predicting the flow of blood donations in Zimbabwe.
- These model results are useful for guiding allocation of blood donation resources and blood donation drive timing.

Blood transfusion is an indispensable therapeutic intervention in any health care system in the care of patients with chronic or other conditions.^1^ However, in other parts of the world, many patients who require transfusion still suffer unnecessarily adverse conditions because of a lack of safe and adequate blood.^2^ The whole blood donation rate per 1,000 population by year is used as an indicator of the general availability of blood in a country.^3^ Many developing countries in sub-Saharan Africa are not able to collect sufficient and safe blood from voluntary blood donors to meet the given benchmark.^2,4^ When coupled with the ever-increasing demand for blood, such low blood donation rates contribute to poor health service delivery and put the lives of many patients at risk with adverse consequences.^5–7^

It is a certainty that blood will be required, but because of unpredictable natural and other manmade accidents and disasters, it is difficult to predict when, where, and what quantities of blood will be required.^8^ Without mathematical or statistical tools to forecast and model blood donation patterns by blood centers, it is difficult for blood managers to be responsive to changes in blood supply. This scarcity of information on annual blood collections has partly made it difficult to accurately determine the demand for blood in some instances.^9,10^ Hence, the collection of data and subsequent modeling and forecasting of blood collections are important for determining blood supply potential.

In Zimbabwe, the National Blood Service Zimbabwe (NBSZ) is responsible for collection, typing into various blood groups, screening, production, and distribution of blood and blood components to hospitals so as to satisfy transfusion demands. To support this mandate, blood donation modeling has become a necessary tool for estimating the supply and demand of blood in the whole blood supply chain.

Zimbabwe faces occasional spikes in both anticipated and unforeseen demand for blood.^11^ The unprecedented spikes in blood demand are a common phenomenon during the public holiday seasons due to increased road accidents, as most people travel during this period. The surge in demand for blood calls for robust approaches to be taken to ensure that the national blood center is adequately stocked to meet routine and emergency demand. Forecasting blood supply is a recurrent challenge for blood center authorities. Reliable forecasts of blood donations are vital for blood managers to manager blood bank inventories.^12–14^

The NBSZ maintains blood stocks at a 5-d supply, which is the recommended optimum level to be maintained. Blood and blood components have a very short shelf life, ranging from 5 days for platelets to 42 days for red blood cells. This perishability leads to blood components being discarded before use and thereby depleting the blood inventory. Therefore, replenishing the blood inventory should be done continuously through blood donations. Any inaccuracy in managing blood donations can have serious ripple effects down the whole blood supply chain, hence the need for continuous monitoring of trends in blood donations and transfusions.^15^

The objectives of the study are to develop time-series statistical models to help in determining optimal blood collection or donation patterns and to use the models in predicting future blood donations. The information gathered from the model can be used by the NBSZ for planning purposes in donor recruitment, blood donation campaign drives, and resource allocation and to prepare the ground for possible future studies in blood supply in Zimbabwe. The model will complement the current approach of relying on collected limited summary statistics in forecasting blood supply and demand in Zimbabwe.

The rest of the article is organized as follows: the next section provides a literature review, and the third section presents the materials and methodology. Data analysis and results are provided in the fourth section. A discussion is given in the fifth section, and the conclusion is presented in the last section.

## Literature Review

There have been numerous studies conducted on the blood supply chain.^16–19^ Several previous time-series forecasting models in the blood supply chain have focused on blood demand and inventory practices to deal with blood components.^14^ However, the focus on forecasting blood donation has not received enough attention compared with other echelons of the blood supply chain, especially in a country such as Zimbabwe. There is generally limited blood supply research literature in sub-Saharan Africa.

A study conducted in New York State of the United States^20^ indicated that an autoregressive moving average (ARMA) model outperformed other time-series forecasting models in predicting future demand of blood. A study to forecast blood supply and demand using demographic data (age and gender) was conducted in Ontario.^21^ The outcome of the predictions showed that the demand for blood products would outpace supply given the age- and gender-specific supply and demand rates at the time of study. However, it was noted that the careful management of the blood supply, together with new medical techniques, could reduce the impact of these future concerns.

A population-based longitudinal study on the implication of demographic changes on blood donation and transfusion demand was carried out in Germany.^22^ The study results showed that blood donation numbers closely followed demographic changes, whereas the demand for transfusion was strongly influenced by changes in medical practice.

Three time-series analysis approaches (i.e., autoregressive [AR] integrated moving average [ARIMA], Holt-Winters exponential smoothing, and neural network–based methods) were employed at a tertiary care hospital in Spain to forecast demand for red blood cells (RBCs).^23^ It was concluded that no single method was superior throughout the different forecasting horizons, and therefore a cocktail of methods was needed to perform the forecasting of the blood demand and fully inform blood bank managers.

In a related study at a tertiary care center in Portugal, time-series prediction models were applied for blood donation inflow forecast.^24^ Six models were developed, namely, AR neural networks, seasonal trend based on locally estimated scatterplot smoothing (loess) with exponential smoothing, Holt-Winters, AR integrated moving average (MA), double-seasonal Holt-Winters, and exponential smoothing state space model with Box-Cox transformation. The study concluded that donation trend lines were better modeled by different models for different forecasting horizons.

Trend models on blood donor and transfusion recipient data based on age and gender categories were also developed in Switzerland.^25^ The study used generalized additive regression and time-series models with exponential smoothing to forecast trends of whole blood donations and red blood cell (RBC) transfusions. The results of the study suggested that the demand for RBCs could equal supply at some point and could eventually cause some blood shortfalls.

Time-series analysis was applied to the donors’ data set collected at a Saudi hospital in Saudi Arabia to establish the presence or absence of seasonal variability.^26^ The study findings showed pronounced seasonality with significant drops in blood donations for the months coinciding with religious festivals. A comparative analysis between time-series methods and machine learning algorithms was conducted for the Taiwan Blood Services Foundation in predicting the supply of blood (RBCs).^27^ The study results indicated that seasonal ARIMA, seasonal exponential smoothing, and multiplicative Holt-Winters models were all good in forecasting the blood supply in the case study data under consideration. It was concluded that there was no single method that could predict the supply of blood accurately at all times. The study further proposed the need to continuously monitor the forecasting accuracy by updating the data\models as information becomes available, thus making the models dynamic and more reliable.

An automated Box and Jenkins seasonal ARIMA (BJ-SARIMA) model to provide models for blood components forecasting was developed in Brazil.^28^ The study concluded that the ARIMA model was an effective and reliable tool in forecasting that helps managers run a blood bank system.

A study in Brazil showed that fluctuation of blood donations did not follow a predictable pattern in blood banks and varied from one blood bank to the other.^29^ The study concluded that such a trend could cause challenges to blood bank managers. The recommendation was that blood banks worldwide should regularly analyze the number of donations and reduce data noise.

The Box and Jenkins approach and the error, trend, and seasonal (ETS) method of time-series analysis will be adopted in this study to produce a reliable forecast using monthly aggregate blood donation data.

By analyzing blood donation patterns in this study using statistical methods, the NBSZ authorities can gain more insights to make informed decisions and interventions to ensure that blood is adequately available in Zimbabwe. The trend in blood donation forecasts determines the availability of blood to meet the future ever-rising demand. The Zimbabwe blood authorities need to strengthen interventions such as marketing, regular awareness campaigns, and donor mobilization to retain old donors and encourage new donors to donate blood.

Findings from the current study will help in providing empirical evidence to sustain the 5-day inventory blood stock cycle maintained by the NBSZ. The weekly donation pattern helps manage the blood stocks to avoid shortages or overstocking. Furthermore, analysis of the daily, weekly, and monthly variations in blood donations can help blood center managers in decision making. Such decisions include allocating blood collection resources, when to conduct the blood donation drives, identifying potential donors to be targeted, and the units of blood to be collected.

## Materials and Methods

This section discusses the sources of data and techniques used in modeling and forecasting blood donations. Blood donations are measured in units of blood. A blood unit is equivalent to a 450-mL blood bag.^30^

### Data Requirements

The blood donations data used are grand totals of blood collections (units) from the 5 regional blood centers in Zimbabwe, namely, Harare, Gweru, Bulawayo, Masvingo, and Mutare. Blood is collected at different times. Blood donation data were collected retrospectively from the NBSZ Laboratory Information Management System and annual reports, which can be downloaded freely from their Web site at https://nbsz.co.zw/, where certain blood donation information is captured in aggregate form. Data on yearly blood donations covering the period 2002 to 2019 were used in the blood analysis of collection trends and formulating a donation index. Data on daily and monthly blood donations for the period from January 2009 to December 2019 were used in the modeling and forecasting. Data between January 2009 and December 2018, giving a total of 120 monthly observations, were used to fit the model, while the data from January 2019 to December 2019 were used in model validation. The study also used data on population growth projections from the Zimbabwe National Statistics Agency’s 2012 Census Population Projections Thematic Report and can be downloaded from their Web site at http://www.zimstat.co.zw/.

### Methods

This study applies time-series models (viz., SARIMA and ETS models) because of their ability to handle seasonal components evident in the blood donations data. Expert opinions and experience were used to make some inferences in the analysis.

The ARIMA methodology used in this study is based on Box and Jenkins.^31^ There are 4 steps to ARIMA model development, namely, model identification, parameter estimation, diagnostic checking, validation and forecasting. The model becomes a SARIMA when the data have a seasonal component. The stationarity of the blood donation series is assessed using the augmented Dickey Fuller (ADF) test. After ensuring stationarity, model identification is conducted using the autocorrelation function (ACF), partial autocorrelation function (PACF), and extended autocorrelation function (EACF). The model parameters are estimated using maximum likelihood estimation (MLE) and diagnostic tests such as the Ljung-Box test, normality plots, Akaike information criterion (AIC), and Bayesian information criterion (BIC). Model performances were evaluated using root mean squared error (RMSE), mean absolute error (MAE), and mean absolute percentage error (MAPE).

Expert opinions and experience were used to make some inferences in the analysis. The time series packages in R version 4.0.2 were used in the data analysis, model fitting, and forecasting. R software was used to create most of the graphs in the study, while Microsoft Excel was applied in the construction of Figures 2 and 6 only.

#### SARIMA model construction

The SARIMA model is generally expressed as *SARIMA (p,d,q)(P,D,Q)s *,^32,33^ where *p*, *d*, and *q* are the nonseasonal AR, no-seasonal differencing, and nonseasonal MA components, respectively. Typically, *P*, *D*, and *Q* denote the seasonal AR, seasonal differencing, and seasonal MA components, respectively, and seasonal *s* denotes the number of seasons.

The SARIMA model for blood donations $\left\{Y_{t\;}\right\}$ can be written as

(1) $$\phi_{p}\left(B\right)\Phi_{P}(B^{s}{)}^{d}(1-B^{s}{)}^{D}Y_{t}=\theta_{q}(B)\Theta_{Q}(B^{s})\varepsilon_{t,}$$

where B is a lag operator defined as $B^{k}Y_{t}=Y_{t-k}$ ; $\varepsilon_{t}$ is the value at time *t* of white noise; ϕ(B) and θ(B) are polynomials of order p and q , respectively; and $\Phi (B^{s})$ and $\Theta (B^{s})$ are polynomials in B of degrees PandQ , respectively.

#### Model identification

A key assumption in the application of SARIMA models to data is that the series must be stationary for the model identification step. The ADF test is a statistical test used to test for stationarity in a time series by determining the presence/absence of a unit root in the series.

The ADF test is expressed as

(2) $$\Delta Y_{t}=\alpha +\delta T+\beta Y_{t-1}+\sum_{i=1}^{k}\phi_{i}\Delta Y_{t-i}+\varepsilon_{t},$$

where $\Delta Y_{t}=Y_{t}-Y_{t-1}$ , *T* is for the deterministic trend, and *$\Delta Y_{t-i}$* is the lagged initial difference to cater for autocorrelation in the data or the error term ( $\varepsilon_{t}$ ). $\phi_{i},\;\delta ,\;\beta$ , and α are model parameters to be estimated.

When the time series data are not stationary, either, say, first-order or second-order differencing and seasonal differencing are applied to the data to make it stationary. The ACF, PACF, and the EACF are used to determine the possible values of p,q,P,andQ .^34^ Several models are initially identified as plausible models.

#### Parameter estimation

The MLE is used to optimize the SARIMA model parameters.^35^

#### Diagnostic checking of the SARIMA model

Residual tests are used to eliminate unqualifying models. These residuals must behave like a white noise sequence if the model is a good fit, with no autocorrelation. The Ljung-Box test^36^ is used to test for this autocorrelation. The ACF/PACF of the residuals are used to see if residuals behave like white noise in the case of the best SARIMA model.

### ETS Method

The ETS model selection entails the identification of the appropriate combinations of the 3 components (error, trend, seasonality). The method uses the weighted average of past observations when forecasting univariate time series.^37^ Various ETS models are generated from combining different seasonal and trend components, namely, additive (A), multiplicative (M), none (N), additive damped (Ad), and multiplicative damped (Md). The best ETS model is determined based on the smallest value of AIC and BIC.^38^

The ETS(A,A,A) model is expressed as

(3) $${\hat{y}}_{t+h|t}=l_{t}+hb_{t}+s_{t+h-m(k+1)}$$

(4) $$y_{t}=l_{t-1}+b_{t-1}+s_{t-m}+\varepsilon_{t}$$

$$l_{t}=l_{t-1}+b_{t-1}+\alpha \varepsilon_{t}$$

$$b_{t}=b_{t-1}+\beta \varepsilon_{t}$$

$$s_{t}=s_{t-m}+\gamma \varepsilon_{t},$$

where

$l_{t}$ is the overall or level smoothing, $b_{t}$ is the trend smoothing, $s_{t}$ is the seasonal smoothing,
${\hat{y}}_{t+h|t}$ is the forecast at h periods ahead, $y_{t}$ is the observation or actual data, t is an index for time period, m is period of the seasonality, k is the integer part of $\frac{\left(h-1\right)}{m}$ , and
$\alpha ,\;\beta^{*},\gamma$ are smoothing parameters (constants) that take a value between 0 and 1 such that the estimation minimizes the error measurements.

$$\varepsilon_{t}=y_{t}-{\hat{y}}_{t|t-1}$$

0<α<1,0<β<αand0<γ<1−α

### Model Validation and Accuracy Measures

The model with smallest value of the goodness-of-fit criteria (i.e., AIC and BIC) was selected as the preferred model.^39^

The model performances are evaluated using 3 performance metrics, namely, RMSE, MAE, and MAPE. Using these 3-performance metrics provides a comprehensive assessment of the accuracy and reliability of the forecasting models. Each performance metric captures different aspects of the forecast error, and using a combination of metrics can help to evaluate the model’s performance across a range of scenarios. RMSE and MAE are absolute measures, while MAPE is a relative measure or scale independent. MAPE returns the error as a percentage, which makes it easier to compare accuracy across data sets.

## Data Analysis and Results

Figure A1 (in the Appendix) shows trends in blood collections and donation index in Zimbabwe for the period 2002 to 2019. The donation index is expressed as donations per 1,000 population and is calculated as follows:

(5) $$\mathrm{DI}=\frac{\left(\mathrm{Units}\;\mathrm{of}\;\mathrm{blood}\;\mathrm{collected}\;\mathrm{per}\;\mathrm{year}\right)}{\left(\mathrm{Total}\;\mathrm{population}\;\mathrm{per}\;\mathrm{year}\right)}\times 1000$$

The donation index follows the pattern of the total blood donations. Throughout the period under study, the blood donation index fluctuated between 3 to 7 donations per 1,000 people, which is close to the World Health Organization guidelines of 6.6 donations per 1,000 people in lower-middle-income countries and 5 donations per 1,000 people in low-income countries, compared with 31.5 donations per 1,000 people in high-income countries.

The blood donation pattern varies from day of the week, month of the year, and year to year in general. Table 1 and the boxplots in Figure 2 depict how blood donations vary by each day of the week during the period 2013 and 2019.

**Table 1:** Summary Statistics of Blood Donations for Each Day of the Week from 2013 to 2019

Day	Monday	Tuesday	Wednesday	Thursday	Friday	Saturday	Sunday
Min	1	1	38	10	3	1	1
Mean	249	267	278	270	249	210	42
Max	612	635	602	685	879	1,165	140

Table 1 summarizes the observations of the number of donations for each day of the week. The results show that the minimum (Min) number of donations per day of the week ranges from 1 to 38 blood units, and the recorded maximum (Max) number of donations per day ranges from 140 to 879 blood units. The mean number of daily donations ranges from 42 to 278 blood units.

**Figure 1:**
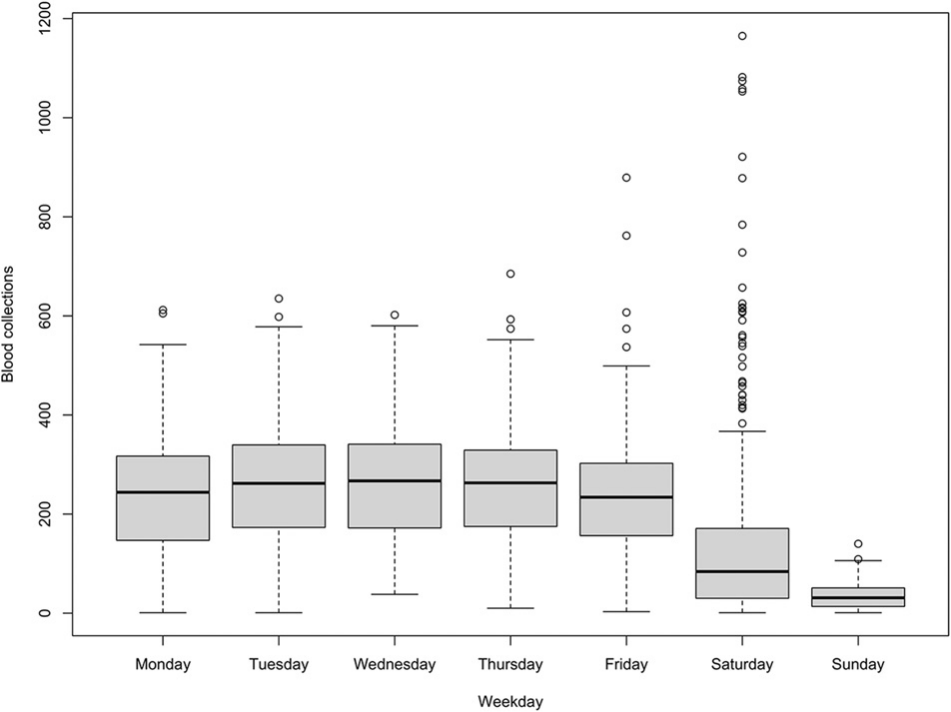
Distribution of blood collections for each day of the week between 2013 and 2019.

The boxplots of blood donations by day of the week in Figure 1 show a near uniform distribution of blood donations from Monday through Friday. Saturdays and Fridays had a number of outliers, with huge donations in some instances. Sundays had the least number of donations with a median of 37 donations, compared with Saturday, which had a median of 132 donations, whereas the other days had medians greater than 240 donations.

**Figure 2:**
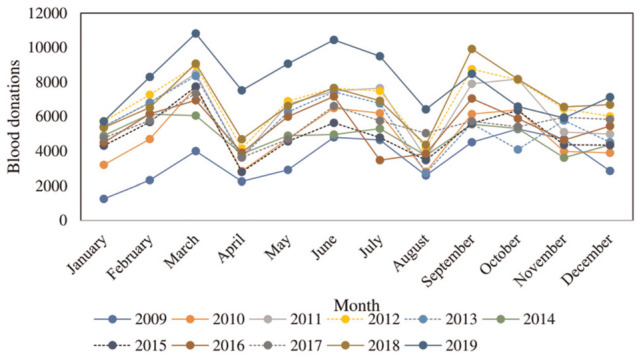
Monthly plot of blood donations for the period 2009 to 2019.

The seasonal plot in Figure 2 and the subseries plot in Figure A2 (in the Appendix) enabled the underlying seasonal pattern to be identified and quantified and any changes in the seasonality over time to be visualized.

Figure 2 shows donations troughs in January, April, August, and December indicating seasonality and is supported by the mean values of blood donations in Figure A2 for April, August, December, and January. The means for the 4 months are much lower than the rest of the months. The seasonal blood donation peaks are in the months of March, June/July, and September.

### Model Identification

**Figure 3:**
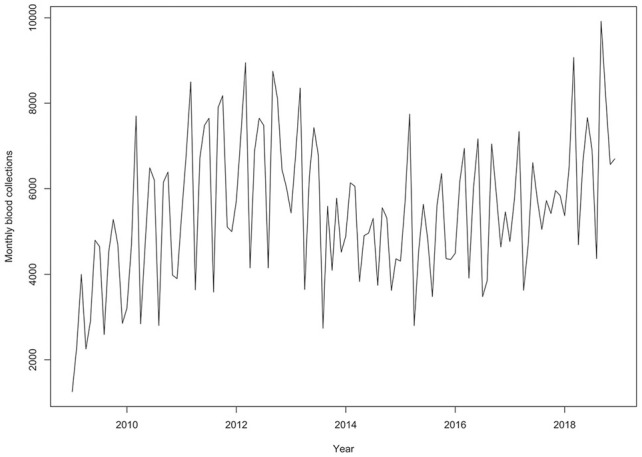
Time-series plot of total blood donations in Zimbabwe from 2009 to 2018.

Data between January 2009 and December 2018 were used to develop the model. A time-series plot of the data is shown in Figure 3.

The time series can probably be described using an additive model, as the fluctuations are slightly constant and less pronounced in size over time and do not seem to depend on the level of the time series.

To see the components of the series, the data were decomposed into the long-term trend, seasonal, and random/residual components as shown in Figure A3 (in the Appendix). The time series is characterized by a weak or slowly varying long-term trend component and a seasonal pattern component. The trend suggests the blood donation series is nonstationary.

The ADF test was used to analyze the stationarity of the original blood donation series. Table 2 gives a summary of the ADF tests conducted and the stationarity conclusions.

**Table 2:** Summary of Augmented Dickey Fuller Tests

Series	Test Statistic	Lag Order	*P* Value	Conclusion
Original series	−1.9101	12	0.61	Nonstationary
Seasonally differenced	−1.5913	12	0.75	Nonstationary
Seasonal and first differenced	−3.8947	12	0.017	Stationary

Based on the significance level of 0.05 and the *P* value of the ADF test =0.61>0.05 , the null hypothesis of a unit root was not rejected, indicating that the original series was not stationary. The presence of a strong seasonality component required the data to be subjected to seasonal differencing first with a period of 12, since monthly data were used.

The ADF test after seasonal differencing gave a *P* value =0.75>0.05 , indicating that the series was still not stationary. The presence of a weak trend in the series led to the application of first ordinary differencing on the seasonally differenced series. The ADF test *P* value =0.017<0.05 confirmed the series was now stationary.

The stationary time-series plot was analyzed further, and the plot of the stationary series is given in Figure A4 (in the Appendix). It is evident from the plot that variance and the trend were stabilized by the differencing, since there is no noticeable change in variation or trend.

**Figure 4:**
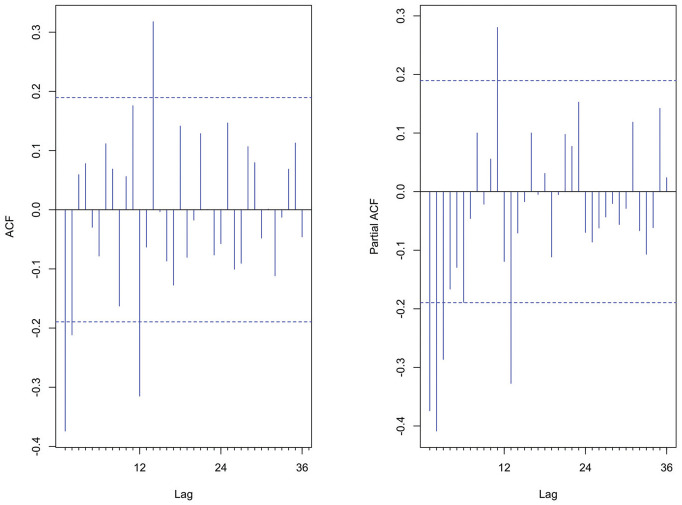
Autocorrelation function and partial autocorrelation function of the stationary series.

The ACF and PACF plots were done to determine the possible values of p,q,P,andQ . Several models were initially identified as possible SARIMA models. Figure 4 portrays the plots of the ACF and PACF used in model identification.

The ACF shows 1 significant spike at lag 1 and another spike slightly above the confidence boundary line at lag 2. This points to a nonseasonal MA (1) or MA (2) process. The PACF shows decaying spikes with significant spikes at lag 1, lag 2, lag 3, and by trial-and-error AR (1) or AR (2) or AR (3) and were considered for further investigation. A significant negative spike at lag 12 on the ACF suggested an SMA (1), while from the PACF there was a significant spike at lag 11, lag 13 and an insignificant spike at lag 12; this might suggest the absence of a seasonal AR model or the possibility of an Seasonal Autoregressive (SAR) (1). From the differencing done above, the nonseasonal and seasonal differences are d=1andD=1 , respectively. Model identification involves so much trial and error. The initial model was set as the $\mathrm{SARIMA}\left(1,1,2\right){\left(0,1,1\right)}_{12}$ model out of so many models evaluated using the time-series packages in R software. An EACF table was used to further confirm the initial model identified.

Table 3 shows the EACF table for the stationary blood donation data after they were subjected to seasonal differencing and first-order differencing. The triangular region of zeros indicates that a mixed model with q=2 and p=0or1or2or3 would be a plausible ARMA (p, q) model. Although the seasonality was at s=12 , significant lags were also found at lags 11 and 13 as a result of the shift caused by differencing. The zero in bold (0) confirms the ARMA(1,2) model component of the $\mathrm{SARIMA}\;\left(1,1,2\right){\left(0,1,1\right)}_{12}$ model.

**Table 3:** Extended Autocorrelation Function Table for the Blood Donation Stationary Data

Autoregressive	Moving Average													
	0	1	2	3	4	5	6	7	8	9	10	11	12	13
0	X	X	0	0	0	0	0	0	0	0	0	X	0	X
1	X	X	**0**	0	0	0	0	0	0	0	0	X	0	X
2	X	X	0	0	0	0	0	0	0	0	0	0	0	X
3	X	X	0	0	0	0	0	0	0	0	0	X	0	X
4	X	0	X	0	0	0	0	0	0	0	0	0	0	X
5	X	X	X	0	0	0	0	0	0	0	0	0	0	X
6	0	X	X	0	0	0	0	0	0	0	0	0	0	0
7	X	X	0	0	0	0	0	0	0	0	0	0	0	0

### Model Fitting

Several possible models were established, and the MLE method was used to make estimates of the parameters. Table A1 in the Appendix gives a summary of the possible models and their parameters.

In Table A1, model 1, the $\mathrm{SARIMA}\;\left(1,1,2\right){\left(0,1,1\right)}_{12}$ model has the lowest Akaike information criterion corrected (for small samples) (AIC, AICc), and BIC values, and all its coefficients were statistically significant with all *P* values being lower than 0.05.

### Diagnostic Checking

**Figure 5:**
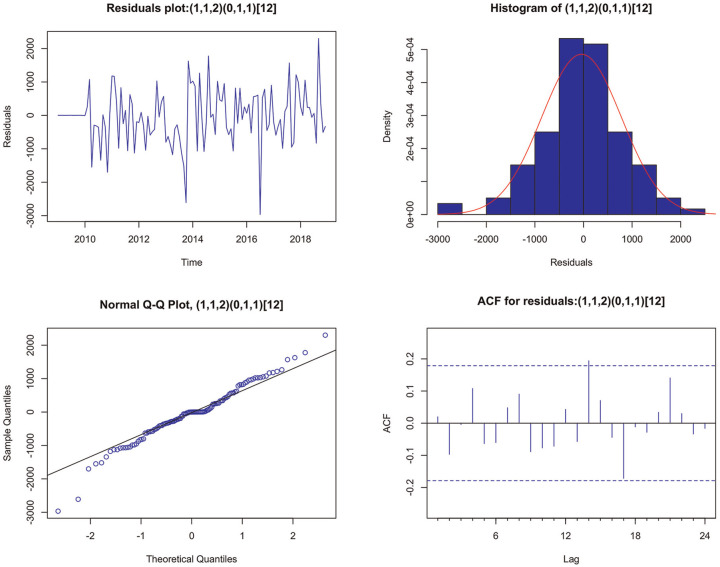
SARIMA (1,1,2) (0,1,1)_12_ model residual plots.

Figure 5 shows the residual plots for the $\mathrm{SARIMA}\;\left(1,1,2\right){\left(0,1,1\right)}_{12}$ model. The residual plot against time shows that the residual errors seem to fluctuate around a mean of zero with a uniform variance. The histogram and Q-Q plot suggested a normal distribution for the residuals. The ACF plot shows that the residuals are independent or not autocorrelated. Even though a slight autocorrelation remains at lag 14 due to random error, the Ljung-Box test for the $\mathrm{SARIMA}\;\left(1,1,2\right){\left(0,1,1\right)}_{12}$ model has a *P* value = 0.33 > 0.05, indicating that the residuals are independent.

### Exponential Smoothing Method

An exponential forecasting method was also used because of its ability to handle seasonality in a series.^32^

#### ETS

An ETS model with additive errors, additive trend, and additive seasonality (A, A, A) that minimized AIC and with the smoothing parameters as α=0.2,β=0.04,γ= 2e-04 was chosen. Furthermore, it had the least absolute MAPE value of 14.30 among the other possible models, as shown in Table 4.

**Table 4:** ETS Models

ETS Model	RMSE	MAE	MAPE	AIC	AICc	BIC
**A, A, A** α=0.2β **= 0.04** γ **= 2e-04**	1523.59	1230.99	14.30	2202.97	2206.97	2241.95
**M, A, M** α **= 0.2609** β **= 0.0168** γ **= 1e-04**	1389.82	1112.34	15.09	2208.02	2214.02	2255.41

AIC, Akaike information criterion; AICc, Akaike information criterion corrected (for small samples); BIC, Bayesian information criterion; ETS, Error, Trend, and Seasonal; MAE, mean absolute error; MAPE, mean percentage error.

### Forecasting and Model Comparison

The selected SARIMA and ETS models were used in generating monthly forecasts of blood collections for the period from 2019 to 2021. The forecasts were used in model cross-validation.

Table 5 shows the accuracy performance measures for the SARIMA (1,1,2) (0,1,1)_12_ and ETS (α = 0.2, β = 0.04, γ = 2e-04) models. There are slight variations in the performance metrics between the SARIMA and ETS models. The SARIMA model fits both the training set and the test set slightly better than the ETS model does. The MAPE values for the test set for SARIMA model (14.38) and ETS model (14.30) are not significantly different. However, based on the results in Table 5 and expert opinion and experience on the blood donation data, the $\mathrm{SARIMA}\;\left(1,1,2\right){\left(0,1,1\right)}_{12}$ model was chosen in this study to model and forecast blood donations in Zimbabwe. The blood center experts have vast knowledge and empirical evidence on blood donation behavior and pattern of the donors.

**Table 5:** Comparison of Models by Performance Measures

	Accuracy Measure	RMSE	MAE	MAPE
SARIMA (1,1,2) (0,1,1)_12_ model	Training set	819.18	599.18	12.13
	Test set	1,449.78	1,218.37	14.38
ETS (α = 0.2, β = 0.04, γ = 2e-04) model	Training set	787.32	623.31	13.63
	Test set	1,523.59	1,230.99	14.30

Table 6 shows the forecasted blood donations for the next 36 mo from January 2019 to December 2021 using the $\mathrm{SARIMA}\;\left(1,1,2\right){\left(0,1,1\right)}_{12}\;\mathrm{model}$ and the ETS (α = 0.2, β = 0.04, γ = 2e-04) model. Confidence intervals (**Lo 95, Hi 95)** are also given, with **Lo 95** as the lower limit of the 95% confidence band for a forecast and **Hi 95** as the upper limit of the same confidence band. The corresponding forecasts between the 2 models are not significantly different. The $\mathrm{SARIMA}\;\left(1,1,2\right){\left(0,1,1\right)}_{12}$ model generated slightly lower forecasts when compared with the ETS (α = 0.2, β = 0.04, γ = 2e-04) model.

**Table 6:** Forecasts of Blood Donations Using the SARIMA and ETS Models

	$\mathrm{SARIMA}\;\left(1,1,2\right){\left(0,1,1\right)}_{12}$ Model Forecast Prediction Interval CI (Lo 95; Hi 95)			ETS (α = 0.2, β = 0.04, γ = 2e-04) Model Forecast Prediction Interval CI (Lo 95; Hi 95)		
Month	2019	2020	2021	2019	2020	2021
January	7,031 (5,294; 8,769)	8,588 (5,566; 11,610)	9,940 (4,559; 15,322)	7,019 (5,362; 8,677)	8,823 (5,584; 12,062)	10,626 (4,263; 16,990)
February	8,431 (6,661; 10,202)	9,966 (6,779; 13,153)	11,306 (5,710; 16,902)	8,448 (6,744; 10,153)	10,251 (6,799; 13,704)	12,055 (5,388; 18,722)
March	10,185 (8,369; 12,001)	11,699 (8,341; 15,058)	13,028 (7,215; 18,841)	10,176 (8,410; 11,944)	11,980 (8,306; 15,655)	13,784 (6,807; 20,761)
April	6,344 (4,469; 8,218)	7,838 (4,302; 11,373)	9,155 (3,123; 15,187)	6,357 (4,512; 8,201)	8,160 (4,253; 12,067)	9,964 (2,671; 17,256)
May	8,251 (6,305; 10,198)	9,727 (6,008; 13,445)	11,033 (4,780; 17,287)	8,374 (6,436; 10,313)	10,177 (6,030; 14,325)	11,981 (4,368; 19,594)
June	9,508 (7,477; 11,540)	10,966 (7,061; 14,870)	12,262 (5,786; 18,738)	9,627 (7,579; 11,676)	11,430 (7,034; 15,827)	13,234 (5,295; 21,174)
July	8,676 (6,548; 10,804)	10,116 (6,021; 14,211)	11,403 (4,703; 18,102)	9,041 (6,866; 11,216)	10,845 (6,191; 15,498)	12,648 (4,376; 20,920)
August	6,842 (4,606; 9,077)	8,265 (3,977; 12,553)	9,543 (2,619; 16,466)	6,937 (4,621; 9,253	8,740 (3,822; 13,658)	10,544 (1,934; 19,153)
September	10,008 (7,655; 12,361)	11,416 (6,932; 15,899)	12,684 (5,536; 19,833)	10,040 (7,570; 12,512)	11,844 (6,654; 17,034)	13,648 (4,695; 22,600)
October	9,501 (7,022; 11,980)	10,894 (6,214; 15,574)	12,154 (4,782; 19,527)	9,792 (7,152; 12,431)	11,595 (6,127; 17,064)	13,399 (4,099; 22,699)
November	8,484 (5,872; 11,096)	9,863 (4,985; 14,741)	11,115 (3,518; 18,712)	8,722 (5,901; 11,542)	10,525 (4,771; 16,279)	12,329 (2,676; 21,982)
December	8,504 (5,752; 11,255)	9,869 (4,792; 14,946)	11,114 (3,293; 18,935)	8,606 (5,592; 11,619)	10,409 (4,363; 16,456)	12,212 (2,202; 22,223)

The forecasted blood donations are believed to be adequate to cater to all blood requirements during the forecasting horizon period. This is under the assumption that the behavior and pattern of blood donors toward donating blood will not change much during the forecasting horizon.

**Figure 6:**
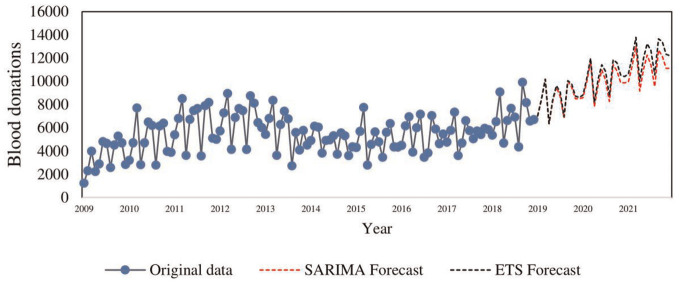
Overlaying forecast plot for SARIMA(1,1,2)×(0,1,1)_12 and ETS (α = 0.2, β = 0.04, γ = 2e-04).

Figure 6 shows an overlaying forecast for blood donations from 2019 to 2021 using the $\mathrm{SARIMA}\left(1,1,2\right){\left(0,1,1\right)}_{12}$ and ETS (α = 0.2, β = 0.04, γ = 2e-04) models. The graph shows an upward trend in blood donation forecasts. The short-term forecasts period (first 24 mo) is consistent with the donation patterns in the preceding years. It is evident from Figure 6 that the $\mathrm{SARIMA}\left(1,1,2\right){\left(0,1,1\right)}_{12}$ model has slightly lower forecasts compared with the ETS (α = 0.2, β = 0.04, γ = 2e-04) model. The $\mathrm{SARIMA}\left(1,1,2\right){\left(0,1,1\right)}_{12}$ model is a versatile technique in short-term forecasting of blood donations.

To further confirm the adequacy of the model in forecasting future blood donations, a paired t test at 5% level of significance was conducted.

The hypothesis being tested was

$$H_{0}:\mu \;(\mathrm{mean}\;\mathrm{difference})=0$$

$$H_{1}:\mu \;(\mathrm{mean}\;\mathrm{difference})\neq 0.$$

A good fit of the model should show no significant differences between the actual and forecasted values. Table 7 shows a summary of the paired t test for such differences in 2019 forecasts.

**Table 7:** Paired *t* Test and CI: Actual Forecast for $\mathrm{SARIMA}\;\left(1,1,2\right){\left(0,1,1\right)}_{12}$ Model

Sample	*n*	Mean	Standard Deviation	Standard Error of the Mean	95% CI for µ (Difference)	*T* Value	*P* Value
Actual	12	7,990	1,720	497	(−1,396, 414)	−1.19	0.257
Forecast	12	8,481	1,235	357			
Paired difference	12	−491	1,425	411			

CI, confidence interval.

From Table 7, a *P* value = 0.257 > 0.05 implied that we cannot conclude that a significant difference exists between the actual and forecasted blood donations. Hence, the model is a good fit.

## Discussion

Historical, validation, and forecast analyses were performed on the series of blood collections data in Zimbabwe. The study included aggregated monthly blood donations data from January 2009 to December 2019. The data were divided into training and test sets. The training data set covered the period from 2009 to 2018, and the test set covered 2019. The out-of-sample forecast was performed for the next 36 months, that is, 2019 to 2021. Expert knowledge of the data and clinical expertise were also used in buttressing the fundamental conclusions made. Several models from different statistical techniques were used, and all proved to be efficient in modeling blood donation.

A plot of the blood donation index together with expert knowledge indicated that the blood supply chain is seasonal and occasionally experiences some volatility as it responds to external and internal shock factors.

The blood donation process modeled in this study exhibited trends ranging from day of the week, month, and seasonal, all spanning across several years. The time-series plots also displayed cyclic behavior. Significant blood donations were made during weekdays and reduced activity experienced over weekends, especially on Sundays. The NBSZ operates a 5-day cycle inventory of blood stocks; hence, a weekly donation pattern helps to manage the blood stocks to avoid shortages or overstocking. Furthermore, analysis of the daily, weekly, and monthly variations in blood donations helps blood center managers made decisions. Such decisions include allocating blood collection resources, when to conduct the blood drives, potential donors to be targeted, and the units of blood to be collected. Resources can be distributed evenly from Monday through Saturday. However, Sunday is not a popular day to collect blood.

The upward trend in the forecasted blood donations ensures the availability of blood to meet an ever-rising demand. This means the blood inventory would be stable in the short- to medium-term forecasting horizons. However, the Zimbabwe blood authorities need to strengthen interventions such as marketing, regular awareness campaigns, and donor mobilization to retain old and encourage new blood donors to donate blood.

Prior expert knowledge about the data suggested the existence of seasonal variation in blood donations mainly during periods associated with public holidays and school holidays in the months of April, August, and December. From that point of view, the seasonality component of the time series was considered to be pivotal in the development of the model, and an idea of a SARIMA model was then pursued.

Seasonal patterns were also observed with significant drops in blood donations in months associated with school holidays and public holidays (April, August, and December/January) due to the reduced number of donors. School and college students are a source of more than 70% of the blood donations in Zimbabwe.^40^ Furthermore, the months from November to early January experience low donations since they are characterized by the festive season, when some businesses scale down on their operations, resulting in reduced to none industry-based blood drives. These cyclic and seasonal trends in blood donation concur with previous findings in other studies.^24,26^ Therefore, the blood center authorities need to conduct blood collection drives prior to this period to ensure the availability of adequate blood during the public holiday period when and where the demand for blood surges.

This study compared the $\mathrm{SARIMA}\left(1,1,2\right){\left(0,1,1\right)}_{12}$ model and the ETS (α = 0.2, β = 0.04, γ = 2e-04) model as parametric models with good forecasting capabilities. The accuracy of performance measures among the models were not significantly different, and this confirmed the conclusion by other researchers that there was no single method that was superior in forecasting in all situations.^23,41^ In other words, a combination of methods was needed to fully forecast the blood donations in different forecasting time periods.

Selection of the best model was done by comparing performance measures, namely, MAPE, RMSE, and MAE. The performance metrics have been used in numerous other studies to measure the accuracy of the models.^42^ Based on the analysis, expert opinion, and experience with blood donation data, the $\mathrm{SARIMA}\;\left(1,1,2\right){\left(0,1,1\right)}_{12}$ model was chosen as the best model in this study to model and forecast blood donations in Zimbabwe. The margins of error would be operationally acceptable, and hence, the proposed forecasting paradigm appeared to yield good estimates for the 12-mo-ahead blood donation forecasts. The model can be constantly updated by applying the latest data available to modify the parameter values.^27^

Since blood donors donate their blood optionally, the uncertainty in blood collection is so high that ordinary nonstatistical techniques may lead to erroneous or unsatisfactory results and decisions. The study findings show the feasibility of blood donation forecasting models in securing the blood supply chain.^43^ With timely and accurate forecasting models, blood managers are able to render better blood supply chain management planning decisions, such as when to collect blood from donors and the quantity of blood units to collect during blood donation drives.^27^

There are some limitations to the study. The forecasting models indicated a continuous increase in future blood donations as the forecasting horizon increases and the confidence intervals become wider. This means that the constructed models are better at forecasting short-term blood donation trends compared with long-term horizons. Therefore, future studies can improve the forecasting accuracy of this study by using other techniques such as deep learning algorithms. There are also no data to support the effects of external factors affecting blood donations, such as economic factors and pandemics. Furthermore, there are no readily available aggregated data on blood demand in Zimbabwe, which are vital for analyzing the blood bank inventory. Data have to be collected from the different blood donation centers.

## Conclusions

This study applied a time-series analysis technique to construct univariate forecasting models for blood donation in Zimbabwe. The SARIMA and ETS models were developed and applied to facilitate the analysis of the historical blood donation trends and seasonality with input from expert prior knowledge of the data. The accuracy performance measures and future blood donation projections were assessed.

The $\mathrm{SARIMA}\left(1,1,2\right){\left(0,1,1\right)}_{12}$ model is a plausible model that can be adopted in the short-term forecasting of blood donations in Zimbabwe. The performance of the SARIMA model showed superior results compared with the ETS performance for the seasonal blood donations data.

This study established that seasonality and trends in blood donation can be incorporated into the prediction/forecasting process using time-series forecasting methods. It is possible to forecast blood donations over short to medium time horizons. Results from the study provide blood center authorities with insights in decision making that help guarantee the availability of blood safety stocks. Decisions such as in the allocation of blood collection resources, when to conduct blood drives, and the expected units of blood to be collected become easier as they are made from an informed position.

## Supplemental Material

sj-docx-1-mpp-10.1177_23814683231222483 – Supplemental material for Application of Time-Series Analysis and Expert Judgment in Modeling and Forecasting Blood Donation Trends in Zimbabwe Click here for additional data file. Supplemental material, sj-docx-1-mpp-10.1177_23814683231222483 for Application of Time-Series Analysis and Expert Judgment in Modeling and Forecasting Blood Donation Trends in Zimbabwe by Coster Chideme and Delson Chikobvu in MDM Policy & Practice
