# Supplementary material for: Application of Time-Series Analysis and Expert Judgment in Modeling and Forecasting Blood Donation Trends in Zimbabwe
Source: MDM Policy Pract. 2024 Jan 18;9(1):23814683231222483. doi: 10.1177/23814683231222483 (PMC10798106; doi:10.1177/23814683231222483)
Supplement: sj-docx-1-mpp-10.1177_23814683231222483 – Supplemental material for Application of Time-Series Analysis and Expert Judgment in Modeling and Forecasting Blood Donation Trends in Zimbabwe [file sj-docx-1-mpp-10.1177_23814683231222483.docx]

**Appendix**


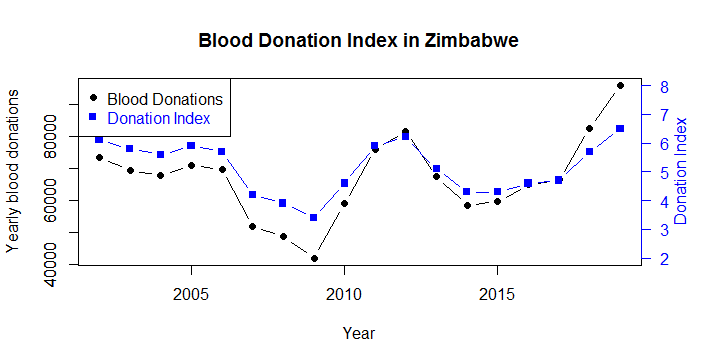


**Figure A1**: Blood trend and donation index in Zimbabwe from 2002-2019

*
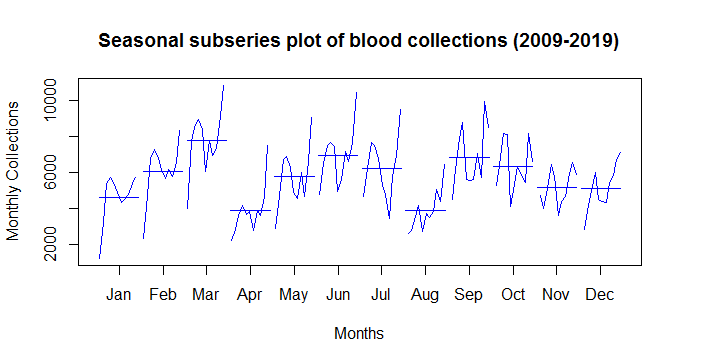
*

**Figure A2**: Seasonal subseries plot of monthly blood donations from 2009-2019


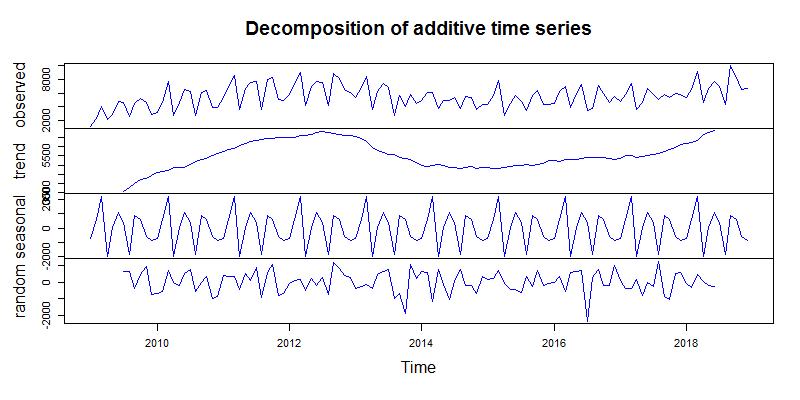
 **FigureA3**: Plot of the decomposed blood donation series (from top: original series, long term trend component, seasonal component, irregular or random/residual component)


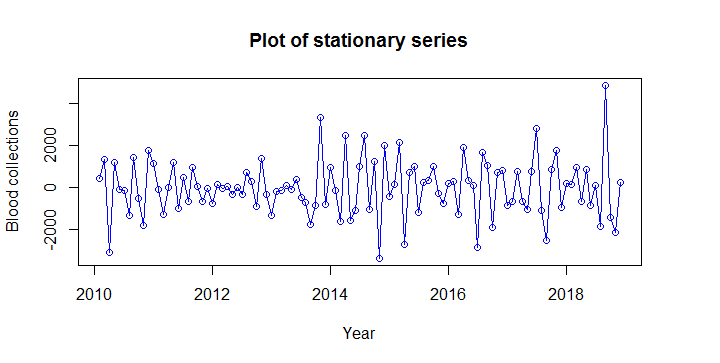


**Figure A4**: Time series plot of the stationary data

**Table A1**: SARIMA models

| Model 1: $\boldsymbol{SARIMA}\left( \boldsymbol{1,1,2} \right)\left( \boldsymbol{0,1,1} \right)_{\boldsymbol{12}}$ model | | | | | | | |
| --- | --- | --- | --- | --- | --- | --- | --- |
| Type | **Coef** | **Coef S.E.** | **Z - value** | **p-value** | **AIC** | **AICc** | **BIC** |
| AR 1 | 0.9531 | 0.0592 | 16.1001 | 2.2e-16 | 1773.81 | 1774.404 | 1787.174 |
| MA 1 | -1.7574 | 0.0710 | -24.7604 | 2.2e-16 |  |  |  |
| MA 2 | 0.8040 | 0.0677 | 11.8826 | 2.2e-16 |  |  |  |
| SMA 1 | -0.8072 | 0.1277 | -6.3215 | 2.591e-10 |  |  |  |
|  | | | | | | | |
| Model 2: $\boldsymbol{SARIMA}\left( \boldsymbol{2,1,1} \right)\left( \boldsymbol{0,1,1} \right)_{\boldsymbol{12}}$model | | | | | | | |
| Type | **Coef** | **Coef S.E.** | **Z- value** | **p-value** | **AIC** | **AICc** | **BIC** |
| AR 1 | -0.1752 | 0.1448 | -1.2099 | 0.22633 | 1778.16 | 1778.751 | 1791.521 |
| AR 2 | -0.2004 | 0.1171 | -1.7110 | 0.08708 |  |  |  |
| MA 1 | -0.5532 | 0.1239 | -4.4630 | 8.081e-06 |  |  |  |
| SMA 1 | -0.7626 | 0.1281 | -5.9543 | 2.612e-09 |  |  |  |
|  | | | | | | | |
| Model 3: $\boldsymbol{SARIMA}\left( \boldsymbol{0,1,1} \right)\left( \boldsymbol{1,1,1} \right)_{\boldsymbol{12}}$ model | | | | | | | |
| Type | **Coef** | **Coef S.E.** | **Z- value** | **p-value** | **AIC** | **AICc** | **BIC** |
| MA 1 | -0.7059 | 0.0605 | -11.6690 | 2.2e-16 | 1777.39 | 1777.785 | 1788.084 |
| SAR 1 | 0.2023 | 0.1158 | 1.7467 | 0.080695 |  |  |  |
| SMA 1 | -0.9999 | 0.3444 | -2.9031 | 0.003695 |  |  |  |
|  | | | | | | | |
| Model 4: $\boldsymbol{SARIMA}\left( \boldsymbol{1,1,2} \right)\left( \boldsymbol{1,1,1} \right)_{\boldsymbol{12}}$ model | | | | | | | |
| Type | **Coef** | **Coef S.E.** | **Z- value** | **p-value** | **AIC** | **AICc** | **BIC** |
| AR 1 | 0.9541 | 0.0680 | 14.0379 | 2e-16 | 1775.56 | 1776.395 | 1791.592 |
| MA 1 | -1.7536 | 0.0715 | -24.5300 | 2e-16 |  |  |  |
| MA 2 | 0.7980 | 0.0682 | 11.7048 | 2e-16 |  |  |  |
| SAR 1 | 0.0925 | 0.1921 | 0.4814 | 0.63020 |  |  |  |
| SMA 1 | -0.9060 | 0.3713 | -2.4404 | 0.01467 |  |  |  |
